# Integrated Clinical Environment Security Analysis Using Reinforcement Learning

**DOI:** 10.3390/bioengineering9060253

**Published:** 2022-06-13

**Authors:** Mariam Ibrahim, Ruba Elhafiz

**Affiliations:** Department of Mechatronics Engineering, German Jordanian University, Amman 11180, Jordan; r.elhafiz@gju.edu.jo

**Keywords:** attack graph, reinforcement learning, artificial intelligence, Integrated Clinical Environment

## Abstract

Many communication standards have been proposed recently and more are being developed as a vision for dynamically composable and interoperable medical equipment. However, few have security systems that are sufficiently extensive or flexible to meet current and future safety requirements. This paper aims to analyze the cybersecurity of the Integrated Clinical Environment (ICE) through the investigation of its attack graph and the application of artificial intelligence techniques that can efficiently demonstrate the subsystems’ vulnerabilities. Attack graphs are widely used for assessing network security. On the other hand, they are typically too huge and sophisticated for security administrators to comprehend and evaluate. Therefore, this paper presents a Q-learning-based attack graph analysis approach in which an attack graph that is generated for the Integrated Clinical Environment system resembles the environment, and the agent is assumed to be the attacker. Q-learning can aid in determining the best route that the attacker can take in order to damage the system as much as possible with the least number of actions. Numeric values will be assigned to the attack graph to better determine the most vulnerable part of the system and suggest this analysis to be further utilized for bigger graphs.

## 1. Introduction

The complexity of networks and security systems, along with the existing vulnerabilities and potential operational faults, necessitates the development and implementation of powerful automated security analysis techniques. These techniques should enable the detection of potential attacks, identification of vulnerabilities, essential network resources, security policies, security bottlenecks, and detecting and correcting faults in network configurations [1].

Different approaches for security analysis, such as qualitative and quantitative risk analysis, can be employed at the design stage. The perspective directions in evaluating the security level of large-scale networks approaches are based on building a representation of the malefactor’s actions in the form of attack trees or attack graphs, checking various properties of these trees or graphs using various methods (for example, model checking), and determining various security metrics [2].

The attack graph technique is a useful method for simulating various attack paths in a network [3]. A quantitative study of attack graph scans reveals crucial information that can assist security practitioners in proactively defending their networks.

In recent years, Artificial Intelligence (AI) has been rapidly applied in various fields, including medical field diagnosis and treatment. In this work, AI will be used to demonstrate the cybersecurity of interoperable medical equipment. An attack graph can be too huge and sophisticated for security administrators to analyze. Thus, Q-learning, a type of model-free reinforcement learning (RL), can be employed to analyze it [4]. In such a scenario, an agent tries one of the many possible actions in a given state and evaluates the results based on the reward he/she receives. The agent repeatedly attempts all potential actions in all states, learning which is the best and making decisions based on a long-term discounted reward [4]. The novelty of this work lies in employing Q-learning techniques to determine the optimal pathway/attack scenario in the attack graph for the Integrated Clinical Environment (ICE) system, depending on the attacker’s reward (for example, the amount of damage an action can cause to the network, and the minimum number of actions that attacker has to make in order to compromise the system). Additionally, this method can be beneficial in determining the most vulnerable part of the system, which can help in building a more secure system. Additionally, our aim is to demonstrate the RL capability in studying the cybersecurity of a physical cyber system. An attacker is assumed to be the agent, and the attack graph developed in our prior work through a case study of ICE [5] is utilized to simulate the environment for Q-learning. Furthermore, the reward system for the attacker’s possible actions is then modelled using the Common Vulnerability Scoring System (CVSS). The Q-learning technique is applied to select the attacker’s possible actions and the optimal path/sequence that the attacker can take to undermine the security of ICE’s network. The remainder of the paper is laid out as follows: In Section 1.1, we look at some of the work that has been conducted in this area. The details of our approach are in Section 2. While in Section 3, we provide the results and analyze them. Finally, in Section 4, our contribution and future work are clarified.

### 1.1. Related Work

#### 1.1.1. RL in the Medical Field

The potential uses of RL in the medical field will be the focus of this section. RL has arisen as a sub-field of AI that studies an agent’s best sequential decision-making process in non-deterministic situations. Given its increasing application to real-world issues such as self-driving vehicles, robotics control, computer vision, bioinformatics, and natural language processing, RL has undergone significant progress in recent years [6].

The authors of [7] suggested that RL has the potential to aid surgical decision making by advising actions at predetermined intervals and its capacity to use complex input data such as text, image, and temporal data in the decision-making process. The algorithm calculates the best recommendation policies by simulating a human trial-and-error learning process. The author discussed the difficulties in the development and application of RL in surgical decision making. Nonetheless, the suggested method overlooks components of medicine that are currently intangible to machines, such as a physician’s perception of a patient and the practice of medicine itself. Patients are highly unique in terms of personality and physiology; thus, there is no such thing as a one-size-fits-all strategy for therapeutic decision making. Instead, a dynamic approach tailored to each patient is required.

Using the RL approach, [8] proposed a new method for medical image segmentation. This unique concept was used to discover the best local thresholding and structuring element values to segment the prostate in ultrasound images. This method can be enhanced in certain ways. For example, states are now defined based on gray-scale values; this should be expanded to include more image properties such as texture and shape. Furthermore, the reward function can be altered to attain complete autonomy. The researchers of [9] constructed a deep reinforcement learning network to predict the location of brain tumors. To demonstrate that RL may assist radiology AI goes past its infancy and into practical use. The researchers trained a deep Q-network (DQN) on 70 post-contrast T1-weighted 2D image slices using the BraTS brain tumor imaging database. They did so in conjunction with picture exploration, using rewards and punishments to pinpoint lesions. As a consequence, RL correctly predicted lesion locations with an accuracy of 85%. The approach can be improved by making its image without the use of an eye tracking point, resulting in a transition from a one-dimensional state and action space to two dimensions and then three dimensions for complete volumetric image stacking.

The authors in [10] developed a robust semantic segmentation technique based on a deep reinforcement learning model. The working idea has been put to the test in a variety of medical photographs. The goal was to address one of the conundrums that are now grabbing the scientific community’s attention. They developed a unique technique to decrease human effort in the extraction of medical picture masks using deep reinforcement learning. A more advanced version of the deep q-learning architecture was introduced. As a result, the method improves deep reinforcement learning for selecting optimal masks during medical image segmentation. Nonetheless, the authors see that the mask extraction stage could be improved in future research.

A reinforcement learning-based antihypertensive medication recommendation system for hypertension patients with type 2 diabetes (T2DM) was proposed in [11]. This study tries to tackle the problem of precision medicine by combining massive amounts of electronic health data with machine learning, which led to the development of Q-learning, a reinforcement learning model. The authors built the model to be as realistic as feasible by integrating hypertension risk factors as a state and antihypertensive medicine as an action. The findings show that the Q-learning model’s hypertension treatment is significant as it correctly predicts the trend of a shift from monotherapy to dual and triple therapy as the patient’s condition worsens. The authors also demonstrated the effectiveness of the proposed methodology by lowering patients’ blood pressure levels. However, they only deal with one type of medical record, which is provided by the Korean national health institution.

#### 1.1.2. RL for Cybersecurity

Cyber vulnerability analysis is defined as “the process of detecting and prioritizing vulnerabilities in a system based on their severity”. It aids in the detection of flaws in a system and the application of appropriate patches [12].

Many pieces of research were conducted to explore RL algorithms to determine the cyber vulnerabilities of different systems and networks. For instance, in [13], an AI-based penetration testing system was suggested and assessed; it employs machine learning techniques, specifically RL, to learn and replicate average and complex penetration testing operations. The suggested system is called the Intelligent Automated Penetration Testing System (IAPTS), and it consists of a module that connects with industrial penetration testing frameworks to allow them to record data, learn from experience, and replicate tests in the future similar testing scenarios. This method attempts to reduce human resources while delivering significantly improved outcomes in terms of testing time, reliability, and frequency. The IAPTS’s main drawback is that it requires high-quality human expert supervision during the early learning phases when a human trainer will perform penetration testing alongside IAPTS, adjust the learning, and veto the system’s output to ensure high-quality training.

An analytical framework is presented by [14] that is made up of five security fields and eight industrial areas in the source. This methodology enables a systematic review of artificial intelligence research that contributes to cybersecurity. The framework is utilized to analyze the patterns and future fields of interest for RL-based research in information system security. However, the proposed framework needs to be refined and validated.

In our previous work [15], we assessed the robustness of power systems in the face of unusual operating conditions, which is critical for adapting successful planning and operation measures. Under sequential topological attacks, the level-of-resilience (LoR) metric was created to assess power system resiliency in terms of the minimal number of faults required to cause a system outage (blackout). The LoR is calculated using four deep reinforcement learning (DRL)-based agents: deep Q-network (DQN), double DQN, REINFORCE (Monte-Carlo policy gradient), and REINFORCE with baseline. Three case studies based on the IEEE 6-bus test system were investigated. The double DQN network agent had the highest success rate and was the fastest among the other agents; accordingly, it can be a useful tool for assessing resiliency. Nonetheless, in this work, we analyze the cybersecurity of the ICE system by utilizing the Q-learning technique to observe the great impact that an attacker may inflict on the system.

A Deep Q-learning-based (DQL) reinforcement learning model to detect and categorize multiple network intrusion attack classes is presented in [16]. A labeled dataset is fed into the proposed DQL model, which subsequently employs a deep reinforcement learning technique based on deep Q networks. The experimental findings showed that the proposed DQL model could learn effectively from the environment on its own and is capable of accurately categorizing different forms of network intrusion attacks. However, the method is not put on a true cloud-based environment to enable the DQL agent to develop its self-learning skills and identify threats with high accuracy in real-time.

## 2. Preliminaries

### 2.1. Attack Graph

Multiple vulnerabilities can be coupled to advance an infiltration using attack graphs. An exploit of vulnerabilities between connected hosts is characterized as a transition between system states in an attack graph, and security-related conditions reflect the system state [17]. There has been already a lot of progress in the generation of attack graphs, with more efficient approaches for doing so [18]. To automatically generate an attack graph, a network attack model is developed first with security conditions and rules (exploits) for modifying the attack state based on the security requirements. The analysis is then carried out to develop exploit sequences that lead to an unsafe network state, which may then be structured in a graph. For this type of network attack analysis, various methods have been proposed, including logic-based (symbolic model checker) approaches [19,20,21] and explicit graph-based approaches [22,23,24].

However, due to their complexity, attack graphs are difficult for humans to use successfully [25]. Even a medium-sized network can contain dozens of possible attack vectors, which can overwhelm a human user with the volume of data given. It’s difficult for a person to figure out which configuration settings should be modified based on the information in the attack graph to effectively address the detected security issues. More effort is also needed to evaluate alternative configuration modifications and verify that optimal changes that are implemented without a good awareness of the existing security problems [26].

Improvements in the visualization of attack paths and the overall presentation of attack graph data have been made in previous work. For example, the researchers in [27] proposed that the usage of protection domains to represent groups of systems with unrestricted interconnectivity can reduce complexity. Visualization approaches were developed in [28] to emphasize essential attack processes while clearly illustrating host-to-host reachability.

**Figure 1:**
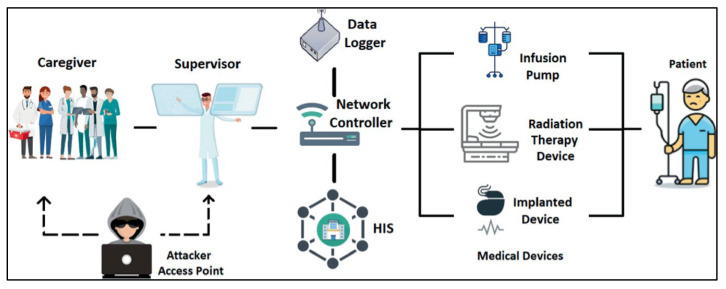
Integrated Clinical Environment (ICE).

**Figure 2:**
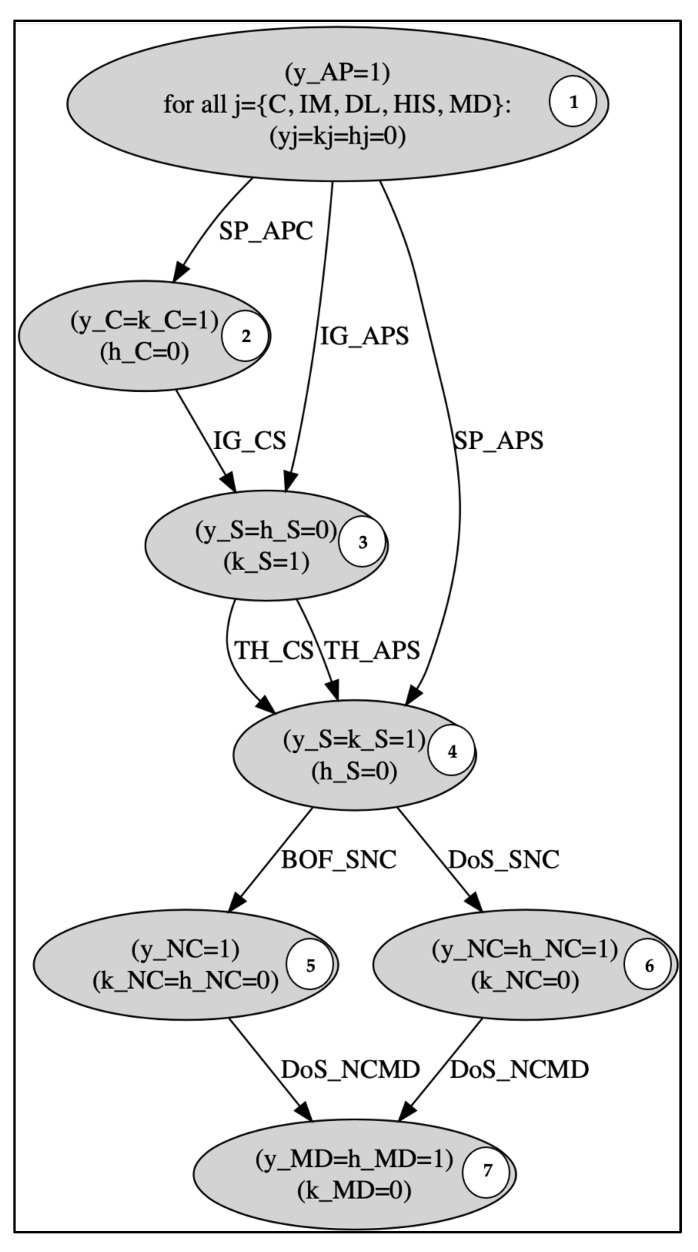
Integrated Clinical Environment’s attack graph.

In this work, the cybersecurity for the ICE system shown in Figure 1 is investigated through the inspection of its generated attack graph from our earlier work [5], as shown in Figure 2.

The ICE system consists of 6 sub-systems: Caregiver (C), Supervisor (S), Hospital Information System (HIS), Data logger (DL), Network Controller (NC) and Medical Devices (MD). The system vulnerabilities that were determined through the network are firmware update vulnerability and Electromagnetic vulnerability (EMV). These vulnerabilities can be exploited producing 5 cyber-attacks: Spoofing (SP), Denial of Service (DoS), Buffer overflow (BOF), Trojan Horse (TH) and Intelligent Gathering (IG). The attack graph that represents how the agent uses the previously mentioned attacks to penetrate the ICE’s network contains 10 different paths and 7 nodes. Each node specifies the evolution of the system’s state whose variables change as a result of attacks (dynamic variables). These variables are: attacker’s authority (y), system information (k), and hardware manipulation (h) of the ICE system components. The components’ inherited vulnerabilities and connectivity (static variables) are necessary as part of the preconditions for attacks to occur.

At the beginning, the attacker is assumed to be at node number 1 and has authority over his/her device, but all the other dynamic variables are assumed to be 0, and the goal is to violate the security property which is presented in the terminating node number 7. The violation of the security property for this system occurs when the attacker has no authority over the MD system and cannot manipulate its hardware. In light of the security property, the attacker’s goal is to gain control and disrupt the medical device’s functionality. He/She can accomplish this by pursuing one of the attack graph’s routes. For example, suppose the attacker has root access to AP at the outset, the attack SP_APS is used to spoof the S’s device. The attacker takes control of the S’s device as well as the S’s authentication credentials. The attacker then does a Buffer overflow attack (BOF_SNC) to seize control of the target NC. Using this permission, a Denial-of-Service attack (DOS_NCMD) attack exploiting EMV in the MD is carried out in order to harm medical devices or drain their batteries, putting the patient’s health at risk.

### 2.2. Common Vulnerability Scoring System

CVSS is an open framework for conveying IT vulnerability characteristics and implications. Adopting this uniform vocabulary will be beneficial for the evaluation of IT vulnerabilities, IT administrators, vulnerability bulletin providers, security companies, application vendors, and researchers [29]. For instance, a graph theory-based security threat model is presented by [30]. They used a Markov chain to discover attack vectors through several security threats in a model with three states for this purpose. To support the demonstration of the security threat model to compute the probability distribution of security threats, twelve security threats were reported by Cloud Security Alliance (CSA), and seven security vulnerabilities rated by CVSS were explored.

The National Vulnerability Database (NVD) and the Exploit Database were utilized by the researchers in [31] to demonstrate how CVSS measures combined with each other can provide better insight into exploit latency. When conditioned on more than two measures, they discovered that there are classes of vulnerabilities with a very short median time to attack (as low as three days). These vulnerability classes give valuable information for prioritizing patching and exploit mitigation.

Nonetheless, the CVSS metric is divided into three categories: Base, Temporal, and Environmental. The Base metric category represents a vulnerability’s inherited properties that remain consistent over time and across user environments. It comprises two types of metrics: those that assess exploitability and those that assess impact. The exploitability metrics represent the ease with which a vulnerability can be abused as well as the technical means by which it can be exploited. That is, they represent characteristics of the vulnerable entity, which is formally referred to as the vulnerable component. The Impact metrics describe the immediate consequence of a successful exploit, and they represent the impact on the thing that is affected, which is formally designated as the impacted component [32].

The Temporal metric category highlights a vulnerability’s features that change over time but not across user environments. The inclusion of a basic exploit kit, for example, would raise the CVSS score, but the creation of an official fix would lower it [33].

The Environmental metric group represents susceptibility characteristics that are specific to a given user’s environment. The presence of security mechanisms that may minimize part or all of the repercussions of a successful attack, as well as the relative importance of a susceptible system within a technology infrastructure, are all factors to consider [34].

In this paper, we used an online CVSS calculator [35] to calculate the CVSS score for each attack in the ICE system, as shown in Table 1.

For instance, for the spoofing attack that was executed by the host AP against subsystem C (SP_APC), the inputs that were fed into the calculator were as follows:

- For the (Attack Vector), our input was Local, which means that the vulnerable part is not linked to the network stack, and the attacker’s direction is via read/write/execute capabilities.
- For the (Attack Complexity), the Low input was fed to the calculator, indicating that there are no special access requirements or mitigating circumstances. When attacking the vulnerable component, an attacker might expect to have consistent success.
- The input for the (Privileges Required) is None, which means that the attacker is unauthorized before preceding the attack, he or she does not need access to the vulnerable system’s settings or data to carry it out.
- Required was the input for the (User Interaction field), meaning that the user must take some action before the vulnerability to be successfully exploited.
- The (Scope) field was answered as Unchanged, indicating that only resources managed by the same security authority can be impacted by an exploited vulnerability.
- The Low answer was fed to the two fields (Confidentiality) and (Integrity), indicating the following: Confidentiality has been compromised, and the attacker has access to some protected information, but he or she has no control over what information is gained or how much of it is obtained. Additionally, for Integrity, it means that the modification of data is possible, but the attacker does not have control over the consequence of a modification, or the amount of modification is limited.
- The (Availability) is None because, within the damaged component, there is no impact on availability.
- (Exploit Code Maturity) input is a Proof-of-Concept, which means for most systems, a proof-of-concept exploit code is available, or an attack demonstration is impractical. The code or technique may not work in all circumstances, and a competent attacker may need to make significant changes.
- Not Defined was the answer for both (Remediation Level) and (Report Confidence); this input shows that there is insufficient information to choose one of the other values and has no effect on the overall Temporal Score.
- For the (Confidentiality Requirement), our input was Medium because the loss of (Confidentiality, Integrity, Availability) will almost certainly have serious consequences on the organization.

These inputs resulted in an overall score of 3.6.

### 2.3. Reinforcement Learning

Quantitative security assessments of large-scale enterprise networks can be performed using Reinforcement Learning (RL) techniques. RL is identified as a type of online learning that takes place in real-time. When interacting with the environment, agents choose and carry out activities that have an impact on the environment. At the same time, agents constantly alter their activities in response to the reinforcement signal provided by the environment by using the trial-and-error approach, and the system behavior obtains the greatest value of the cumulative reward from the environment [36].

Q-learning [4] is one of the RL strategies. It is a simple method for agents to carry out actions optimally in controlled Markovian domains [37]. Its model consists of an agent, states, and a set of actions for each state for the agent. An action refers to the agent’s transition between states. The agent receives a quantitative value reward for taking an action in a certain state. The agent’s goal is to maximize his/her entire reward. The associated reward, which is environmental feedback, determines the quality of each action [38].

A novel Q-learning-based vulnerability study of the electrical power grid in sequential topological attacks was described in [39]. The Q-learning-based sequential method was able to identify vulnerable sequences that led to severe blackouts in the system by monitoring the change in the system’s topology. This technique not only increased the number of line outages through the learning process but also reduced the number of attacks initiated by removing failed attack sequences that did not exploit the cascading outage vulnerability.

The authors of [40] provided a game-theoretic approach for describing the decision-making process of cybersecurity monitoring. They examined versions of Q-learning algorithms to reflect the genuine conditions of decision making in a security game (e.g., Minmax and Naive Q-learning and Markov games).

Q-learning is a straightforward method for agents to take optimal actions in controlled Markovian domains [41]. Since this model seeks to find an optimal action selection policy through agent learning that maximizes the sum of reinforcement functions, rational allocation aids in learning efficiency. The most common type of reinforcement signal is a scalar, which is commonly stated as a positive encouragement or a negative punishment. The agent’s living environment is described as a set of possible states S, in which the agent may perform a likely action *a* within a set of possible actions A, then the agent earns a real return *r*. The aim of the agent is to learn a control strategy (Optimal policy π) π:S→A to maximize the expected value of these returns, with the subsequent return value lowered as the index delay increases [42]. To learn Q-value, Equation (1) describes a reliable way to estimate the training value on the basis of immediate return sequences expanded in the timeline only [43].

(1) $$\begin{array}{c} Q\left(s,a\right)=r\left(s,a\right)+\gamma maxQ\left(\delta \left(s,a\right),\overset{´}{a}\right)\;\\ \overset{´}{a} \end{array}$$

where γ represents a discount factor that is set between 0 and 1, which models the fact that future rewards are worth less than immediate rewards; in this work, the value of γ is 1. $\delta \left(s,a\right)$ is the new state resulting from applying action a to s.

The learning machine represents assumptions via a Q-table in the training algorithm, with each state–action pair having an entry that stores the current assumptions of $Q\left(s,a\right)$. The agent observes the current state s, selects and performs a specific action a, and then observes the returned result $r\;=\;r\left(s,a\right)$ and the new state $s\prime =\;\delta \left(s,a\right)$. The agent then updates each of these entries. Equation (2) represents the rules [44]:


(2) $$\begin{array}{c} \hat{Q}\left(s,a\right)\leftarrow r+\gamma \max\hat{Q}\left(\overset{´}{s},\overset{´}{a}\right)\\ \;\;\;\;\;\;\;\;\;\;\;\;\;\;\;\overset{´}{a} \end{array}\;$$

The agent was utilized in this training process to refine the previous state estimate using the current Q value of the new state. The agent travels through states till he/she reaches the aimed destination (termination state). An episode is a journey that begins with the initial state and finishes with the goal state. When the agent reaches the objective state, he/she begins the next episode [44].

The calculation of the Q table is based on a single goal, and in our work, the goal is to reach a state where the security property is violated, which is presented only in the termination node number 7.

Our used approach is provided in Algorithms 1 and 2, respectively. Initially, the attack graph is generated for the ICE system using our earlier work [5] based on Architecture Analysis and Design Language (AADL) and JKind model checker with Graphviz visualization tool. Next, the attack graph is refined using the CVSS overall scores to assign the rewards values with the RL environment constituting a refinement graph. The Q-learning technique is applied to select the attacker’s possible actions and the optimal path/sequence that the attacker (agent) can take to undermine the security of ICE’s network.

Markov Decision Process (MDP) is a stochastic control mechanism that operates in discrete time. It is a collection of alternative states that a system can be in. The Reset function is used to return the agent to its initial state, which is specified as state number 1. This function is called at the start of each training episode and simulation. The terminal state is used to describe the goal state (node number 7). The Sim function is a function that is used to simulate a reinforcement learning environment with an agent configured for that environment. The transition matrix is the likelihood that an action taken in stat s will result in a transition to state s′. A transition matrix describes the probability of a successful attack in an attack scenario.
**Algorithm 1.** Used Approach**Result:** Best Solution (Route) and Cumulative Reward Initialization;1. Generate Attack Graph Using Architecture Analysis and Design Language, JKind checker tool and Graphviz2. Convert Attack Graph to Refinement Graph;3. Formulate the RL problem. Define environment, agent, states, actions, and rewards;4. Train RL Agent in MDP Environment;

**Algorithm 2.** Train RL Agent in MDP Environment**Result:** The Agent Successfully Finds The Optimal Path Which Results In Cumulative RewardInitialization;Create MDP Environment;1. Create MDP Model With Identified States And Actions;2. Specify The State Transition And Reward Matrices For The MDP;3. Specify The Terminal States Of The MDP;4. Create The RL MDP Environment For This Process Model;5. Specify The Initial State Of The Agent By Specifying A Reset Function;Create Q-Learning Agent;1. Create A Q Table Using The Observation And Action Specifications From The MDP Environment;2. Set The Learning Rate Of The Representation;3. Create A Q-learning Agent;Train Q-Learning Agent;1. Specify The Training Options (Episode, Stop Training Criteria);2. Train The Agent Using The ‘train’ Function;Validate Q-Learning Results;1. Simulate The Agent In The Training Environment Using The ‘sim’ Function.

**Figure 3:**
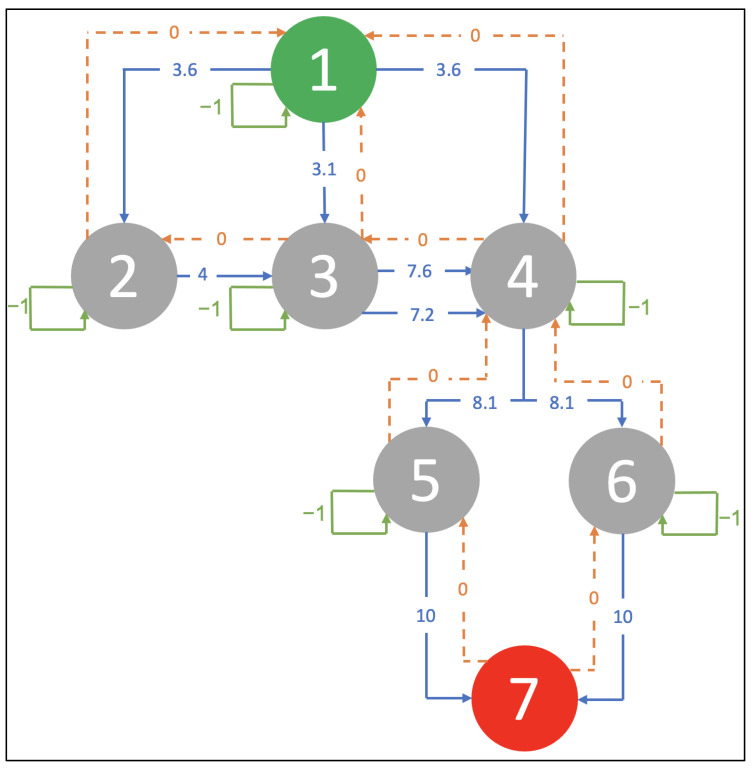
Refinement graph with nodes from 1–7 representing systems’ states as given in attack graph.

Figure 3 demonstrates a refined graph that presents the Q-learning environment along with the reward values. These values are assigned to each action that the agent may select. Vertices (states) represent the system evaluation of dynamic parameters.

At the beginning, the agent location is assumed to be at node number1 (green node), and he/she can move between the nodes in any path until he/she arrives at the goal state (node number 7 (red node)). Furthermore, the CVSS overall scores are used to assign the rewards values. When the attacker reaches the goal state, he/she will stay there forever, and the cumulative reward will be the sum of each action’s reward. If the agent takes a backward move or action, the reward for it will be 0. If the agent does not move (stays in the same node) or moves to a node that is not connected to the node that he/she is in, the reward will be −1.

Table 2 summarizes the possible rewards the agent obtains when moving among the seven nodes. For example: if the attacker moved from node number 2 to node number 3, the reward will be 4.

## 3. Results and Discussion

In this section, we used Python language to apply our Q-learning technique to determine the worst-case attack scenario an attacker can execute against the ICE system, as determined by the maximum cumulative reward with the least number of actions to damage the system. The execution time for the used technique is less than an hour on a standard computer processor: 2.3 GHz 8-Core Intel Core i9; memory: 16 GB, 2667 MHz DDR4, running macOS Big Sur.

**Figure 4:**
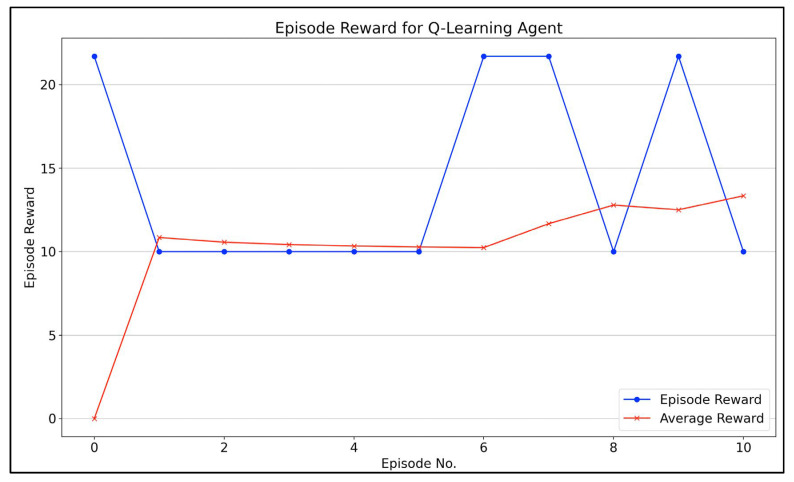
Results of Q-learning agent.

Figure 4 demonstrates the agent’s training progress when finding the attacker’s/agent’s best route. The x-axis illustrates the number of episodes, and the y-axis illustrates the cumulative reword for every episode. It took around 10 episodes for the model to converge. The blue line illustrates the cumulative reward for each episode, while the red one shows the change in the average reward after each episode, indicating the change in the agent training after each episode. In other words, we can see in the figure that the average reward is increasing, which means that the agent’s training is improving throughout the episodes.

The results shows that the worst attack scenario route is through nodes: 1⟶4⟶6⟶7, with a cumulative reward of 21. Using this information, we can observe the maximum damage the attacker can cause, and we can rank the vulnerable subsystems accordingly. Noticing that the first attack was from node number 1 to node number 4. From the attack graph, we can determine the attack destination is the Supervisor. Then, the agent moved from node number 4 to node number 6, which means that the agent attacked the Network Controller. Finally, he/she attacked the Medical Devices by moving from node number 6 to node number 7. Thus, the most vulnerable subsystems are the Supervisor, Network Controller, and Medical Devices. We can also rank the most to the least vulnerable subsystem using the CVSS metric. The most vulnerable subsystems are Medical Devices, then the Network Controller, and finally, the Supervisor, and this can be concluded by comparing the CVSS value for each attack that was executed against each subsystem and observing the diversity of the values. For instance, the CVSS value for the attack that was executed against the Medical Devices is 10, which is the largest number on the CVSS scale, while the CVSS values for the attacks that were executed against the Supervisor differ from 3.1 to 7.6, which are less than 10, and this indicates that the Supervisor is not as vulnerable as Medical Devices.

The authors of [45] used multi-host multi-stage vulnerability analysis (MulVAL) hypothetical analysis to determine all attack scenarios (attack graph) of a network as opposed to our earlier work based on AADL and JKind model checker [5]. The MulVAL cannot examine confidentiality or integrity losses vulnerabilities (privilege escalation and denial of service) as opposed to our attack modeling tool that can examine confidentiality, integrity, and availability attacks. They refined the attack graph to generate a transition graph to model the environment for Q-learning along with CVSS scores for the attacker’s actions. The generation of the transition graph requires four processes: Process 1 generates all the paths between attack goals and leaves, which have vulnerabilities. Process 2 generates edges between leaf nodes that have a vulnerability, and it also gives paths that contain nodes with an edge to the attack goals. Process 3 finds edges to the attack goals. Process 4 determines the attacker’s initial potential actions and related edges for the transition graph. However, in our work, the refined graph is just based on assigning reward values (CVSS scores) immediately to the attack graph transitions among nodes representing the Q-learning environment.

Different applications of RL that would assist in supplying efficient decisions for enhancing patient health treatment, prognosis, diagnosis, and condition are discussed in [46]. RL concentrates on long-term rewards, and it is also capable of managing long and complicated consecutive decision-making duties with sampled, delayed, and exhaustive feedback. Our proposed Q-learning approach can be also applied in many real cyber physical systems applications for security analysis, such as smart grids, wastewater treatment, nuclear power plants, communication networks, and smart homes.

## 4. Conclusions

This work represents an attempt to address the most critical aspect of designing secure ICE systems, which is their cyber-vulnerability through a unique approach based on the application of Q-learning to the attack graph, which was generated to sum the possible attack scenarios performed against the ICE. Firstly, a refinement graph was employed to model the environment, which is a simplified attack graph. Then, the CVSS metric was used to assign rewards to each state transition. Afterward, the RL problem was formulated, and finally, the agent was trained in an MDP environment, where the agent successfully found the optimal path. In our findings, the most vulnerable subsystem was identified along the optimal path that an attacker could take in order to harm the system with the least number of steps. These findings can aid with the creation of the optimal action selection rules to patch the vulnerabilities. In the future, this approach can be enhanced by introducing the defender that makes appropriate preemptive responses based on a limited view of the system condition, which is achieved through the use of monitors. Additionally, it can be further experienced on bigger-sized attack graphs. Additionally, other techniques can be used to study ICE’s cybersecurity, such as deep reinforcement learning and SARSA learning.

## Figures and Tables

**Table 1:** Attacks’ CVSS Scores.

Attack Name	Base Score	Temporal Score	Environmental Score	Overall Score
SP_APC	4.4	4.2	3.6	3.6
IG_APS	3.5	3.1	3.1	3.1
SP_APS	4.4	4.2	3.6	3.6
IG_CS	3.5	3	4	4
TH_CS	8	7.5	7.6	7.6
TH_APS	7.6	7.1	7.2	7.2
BOF_SNC	8	8.1	8.1	8.1
DoS_SNC	8	8	8.1	8.1
DoS_NCMD	7.5	7.5	10	10

**Table 2:** Reward matrix.

R	1	2	3	4	5	6	7
1	−1	3.6	3.1	3.6	−1	−1	−1
2	0	−1	4	−1	−1	−1	−1
3	0	0	−1	7.6	−1	−1	−1
3	0	0	−1	7.2	−1	−1	−1
4	0	−1	0	−1	8.1	8.1	−1
5	−1	−1	−1	0	−1	−1	10
6	−1	−1	−1	0	−1	−1	10
7	−1	−1	−1	−1	0	0	−1

## Data Availability

Data is contained within the article.
